# High-frequency irreversible electroporation improves survival and immune cell infiltration in rodents with malignant gliomas

**DOI:** 10.3389/fonc.2023.1171278

**Published:** 2023-05-05

**Authors:** Sabrina N. Campelo, Melvin F. Lorenzo, Brittanie Partridge, Nastaran Alinezhadbalalami, Yukitaka Kani, Josefa Garcia, Sofie Saunier, Sean C. Thomas, Jonathan Hinckley, Scott S. Verbridge, Rafael V. Davalos, John H. Rossmeisl

**Affiliations:** ^1^ Bioelectromechanical Systems Laboratory, Virginia Tech, Blacksburg, VA, United States; ^2^ School of Biomedical Engineering and Sciences, Virginia Tech-Wake Forest University, Blacksburg, VA, United States; ^3^ Department of Small Animal Clinical Sciences, Virginia Tech, Blacksburg, VA, United States

**Keywords:** glioblastoma, intracranial, electroporation, blood-brain barrier disruption, immune response, numerical modeling, pulsed electric field (PEF)

## Abstract

**Background:**

Irreversible electroporation (IRE) has been previously investigated in preclinical trials as a treatment for intracranial malignancies. Here, we investigate next generation high-frequency irreversible electroporation (H-FIRE), as both a monotherapy and a combinatorial therapy, for the treatment of malignant gliomas.

**Methods:**

Hydrogel tissue scaffolds and numerical modeling were used to inform *in-vivo* H-FIRE pulsing parameters for our orthotopic tumor-bearing glioma model. Fischer rats were separated into five treatment cohorts including high-dose H-FIRE (1750V/cm), low-dose H-FIRE (600V/cm), combinatorial high-dose H-FIRE + liposomal doxorubicin, low-dose H-FIRE + liposomal doxorubicin, and standalone liposomal doxorubicin groups. Cohorts were compared against a standalone tumor-bearing sham group which received no therapeutic intervention. To further enhance the translational value of our work, we characterize the local and systemic immune responses to intracranial H-FIRE at the study timepoint.

**Results:**

The median survival for each cohort are as follows: 31 days (high-dose H-FIRE), 38 days (low-dose H-FIRE), 37.5 days (high-dose H-FIRE + liposomal doxorubicin), 27 days (low-dose H-FIRE + liposomal doxorubicin), 20 days (liposomal doxorubicin), and 26 days (sham). A statistically greater overall survival fraction was noted in the high-dose H-FIRE + liposomal doxorubicin (50%, p = 0.044), high-dose H-FIRE (28.6%, p = 0.034), and the low-dose H-FIRE (20%, p = 0.0214) compared to the sham control (0%). Compared to sham controls, brain sections of rats treated with H-FIRE demonstrated significant increases in IHC scores for CD3+ T-cells (p = 0.0014), CD79a+ B-cells (p = 0.01), IBA-1+ dendritic cells/microglia (p = 0.04), CD8+ cytotoxic T-cells (p = 0.0004), and CD86+ M1 macrophages (p = 0.01).

**Conclusions:**

H-FIRE may be used as both a monotherapy and a combinatorial therapy to improve survival in the treatment of malignant gliomas while also promoting the presence of infiltrative immune cells.

## 1 Introduction

The most common and aggressive malignant brain tumor, glioblastoma (GBM), demonstrates a 5-year survival rate of only 6.8% (1). Notable difficulties arising from efficacious GBM treatment include: (i) an inability of current standard of care to target highly invasive GBM cells which have migrated beyond the visible tumor into healthy brain parenchyma; (ii) limitations in delivering therapeutic agents to diseased regions in the brain, due in part to the impeditive functions of the blood-brain barrier (BBB); and (iii) gross, microscopic, and genetic intratumor heterogeneity, complicating molecular targeting of the GBM tumor (2–6). A significant advancement in the management of GBM arose from the introduction of the chemotherapeutic agent temozolomide to the standard of care, extending median overall survival from ~12.1 months to ~15 months (7). Despite these promising results, GBM remains a highly dismal prognosis.

Standard of care for GBM consists of tumor debulking with surgical resection, followed by radiation therapy and concomitant and adjuvant temozolomide (8). Two proposed alternate therapies include tumor treating fields (TTFields) and laser interstitial thermal therapy (LITT). TTFields is a non-invasive therapy which uses scalp electrodes to induce a dielectrophoretic force on GBM cells and inhibits cell division (9). A randomized clinical trial in patients undergoing either maintenance temozolomide or TTFields with maintenance temozolomide showed the TTFields-treated group had an increased median overall survival (16 vs. 20.9 months, respectively) (10). TTFields has significantly impacted the management of GBM, though further investigation of this therapy is warranted as implementing TTFields currently requires high patient adherence to wearing scalp electrodes >18 hr per day, which can lead to secondary effects such as contact dermatitis (11). Secondly, LITT is a minimally invasive thermal therapy utilizing catheter-based, MRI-guided laser heating to ablate GBM tumor tissue. Laser heating (either 90 vs 1064 nm) is used to induce focal hyperthermia and intraoperative real-time feedback of treatment progression is acquired using either a thermocouple or MRI-thermometry (12). LITT is currently under clinical investigation and shows promise in treating tumors near sensitive structures where surgery is not amenable (13–15). It should be noted that the diffuse thermal energy deposited during LITT may inadvertently damage eloquent structures by causing charring and carbonization of tissues adjacent to the tip, and that MRI thermometry is currently not achievable adjacent to bone.

These drawbacks highlight the need for an alternative approach for tumor cytoreduction in conjunction with enhanced peritumoral drug delivery. To this end, electroporation-based therapies have previously been investigated for intracranial applications, including CNS tumor ablation and transient disruption of the BBB (16–18). Electroporation therapies use high amplitude pulsed electric fields to exogenously raise the transmembrane potential of a cell above a critical threshold, leading to formation of defects on the cell membrane (19). These defects facilitate an increase in cell membrane permeability and are suitable for applications in enhanced delivery of plasmid DNA (electrogene-transfer (EGT)), enhanced delivery of molecular adjuvants and chemotherapeutic reagents (electrochemotherapy (ECT)), induction of non-thermal cell death (irreversible electroporation (IRE)), and for tumor immunomodulation (20–22). IRE occurs when the applied electric field is beyond the cell death threshold. It has been suggested that the non-thermal cell death mechanism induced by IRE can improve upon the antigen presentation and consequently the immune response (23). The anti-tumor effects of IRE are improved by, but not dependent upon, its immunomodulatory outcomes.

Monophasic pulses of around 100 microseconds are commonly delivered in IRE treatments. Despite its promising outcomes, IRE treatments are associated with pain and muscle contractions. Thus, high-frequency irreversible electroporation (H-FIRE), a second-generation IRE treatment protocol, was developed to mitigate the limitations of IRE. H-FIRE is administered intracranially by inserting needle-electrodes through a burr hole craniectomy, where insulation along the electrode ensures only the target tissue is exposed to high-magnitude electric fields (24). Notable benefits of intracranial H-FIRE ablation include: (i) non-thermal cell death, where protein structures in the ablation region are spared; (ii) peri-ablative disruption of the BBB for a duration up to 72 hours post-treatment; (iii) sharp ablative boundary, with a sub-millimeter delineation between ablated and intact tissue; (iv) enhanced susceptibility of cancer cells to H-FIRE ablation *in vitro*; (v) cell death is in part pro-inflammatory (necrosis and pyroptosis), producing antigens that modulate and recruit the immune system; (vi) treatment is not significantly influenced by neighboring anatomical structures, enabling tumor ablation near eloquent anatomies, such as the skull, ventricles, or vasculature in the brain (25–29). Unlike traditional IRE, H-FIRE utilizes bursts of biphasic pulsed electric fields to non-thermally ablate neoplastic and non-neoplastic tissue while mitigating excitation of skeletal muscle and nerves during tissue ablation. Additionally, the utilization of bipolar pulses results in a net charge delivery of zero contributing to a reduction of electrolysis which when present, produces unwanted electrochemical effects including pH changes which may affect the cellular response to treatment (25).

Several electroporation-based treatments have been utilized for treating preclinical models of GBM. The safety and technical feasibility of intracranial electroporation has been established, though few efficacy studies have been conducted (30). Sharabi et al. (31) recently investigated the preclinical efficacy of ECT, a non-ablative electroporation modality used to focally increase the cytotoxicity of molecular adjuvants, for treatment of rodent glioblastoma. It was demonstrated ECT (cisplatin + electroporation) extended median survival from 15 to 22 days, compared to the cisplatin only group (31). Rossmeisl et al. investigated first generation IRE therapy for in situ, non-thermal ablation of high-grade glioma in canine patients presenting with spontaneous brain tumors. The median 14-day post-IRE Karnofsky Performance Score of subjects surviving to discharge (n=6/7) was improved over pre-treatment values in all cases by an average 16 points (32). Agerholm-Larsen et al. investigated preclinical efficacy of ECT (electroporation + bleomycin) for treatment of N32 glioma in rodents. Similarly, there was a significant improvement in survival (P<0.001) between the ECT group and the comparison group (electroporation only, bleomycin only, or no treatment) (33). These studies support intracranial application of electroporation, with improved neurocognitive outcomes.

To build upon preclinical studies of first-generation electroporation-based therapies for brain malignancies, here, we investigate the efficacy of both monotherapy and combinatorial (liposomal doxorubicin) H-FIRE for the management of GBM. To further develop our understanding of tumor response to H-FIRE, we briefly investigate the recruitment of the immune system post H-FIRE treatment. This survival endpoint study utilizes an orthotopic F98 rodent glioma model and survival is conducted against a sham, no treatment group.

## 2 Material and methods

### 2.1 Bioluminescent F98 rodent glioma cell lines

Our orthotopic tumor model comprises the Fischer rat strain and the highly infiltrative Rattus norvegicus F98 undifferentiated malignant glioma cell line (certified pathogen free, ATCC, Manassas, VA). The F98 glioma model was selected as it shares many characteristics with human GBM gliomas including an infiltrative growth pattern. Prior to implantation, F98 cells were transfected to express a plasmid coding for red-shifted firefly luciferase (pLL-EF1a-Luciferase-T2A-Puro Lenti-Labeler Lentivector Virus, Systems Biosciences Incorporated) for bioluminescent imaging allowing for tumor growth progression. Cells were maintained using conventional cell culture technique and prepped for both *in vitro* and *in vivo* investigations. Briefly, F98 cells were maintained at 5% CO2 and 37°C in Dulbecco’s Modified Eagle Medium (ATCC) supplemented with 1% penicillin/streptomycin (Life Technologies), and 10% fetal bovine serum (R&D systems). Cells were routinely passaged at 70-90% confluence.

### 2.2 Assurances and surgical procedures

The study was performed in accordance with the principles of Guide for the Care and Use of Laboratory Animals and was approved by the Institutional Animal Care and Use Committee (IACUC #19-217). Study animals were adult male Fischer rats, weighing between 200-225g at the time of treatment. Following a 2-week acclimation period, rodents were premedicated with a subcutaneous (1mg/kg) injection of buprenorphine (Ethiqa XR. Fidelis Pharmaceuticals, North Brunswick, NJ), anesthetized using isoflurane induction (3-4%: 95% isoflurane: oxygen mixture), and then maintained with isoflurane (2-3.5%: 95% isoflurane: oxygen mixture) delivered *via* nosecone. The dorsum of the head from the intercanthal area to the cranial cervical region was clipped and prepared for aseptic surgery. Anesthetized rats were instrumented in a small animal stereotactic headframe (Model 1350M; David Kopf Instruments, Tujunga, CA, USA). A unilateral rostrotentorial surgical approach to the skull was performed and a 4 mm x 2.5 mm rectangular, parietal craniectomy defect was created in the skull of each rodent using a high-speed electric drill (Dremel 3000 Series; Mount Prospect IL, USA) with a 2.4 mm diameter, round burr.

Using the newly formed craniectomy defect, F98 malignant glioma cells were implanted using sterile, stereotactic surgery. A total of 10,000 cells in 5 μL of phosphate-buffered saline (PBS) were injected into the brain using a 29-gauge needle during a period of 2 minutes to prevent efflux. After the injection, the needle was kept in place for 1 minute, and then slowly retracted to prevent the spread of tumor cells. The incision was closed, and therefore after reopened at the 7-day timepoint (as determined during the tumor growth study), in preparation for H-FIRE treatment. Following H-FIRE pulse delivery, the electrodes were retracted, the craniectomy defect covered with bone wax (Ethicon), and the skin incision closed with 4-0 Monocryl interrupted skin sutures (Ethicon, Somerville, NJ, USA). Rats were recovered from anesthesia and monitored until their predetermined survival endpoints.

### 2.3 Characterization of the bioluminescence kinetic curve and tumor growth study

Bioluminescent imaging with *In Vivo* Imaging System (IVIS) (Perkin Elmer), is understood to be a transient process. Therefore, a standard kinetic curve was developed to determine an optimal time window for characterization of the luminescent signal. Rodents were anesthetized and administered an intraperitoneal 30 µg/ml (150 mg/kg body weight) injection of D-luciferin (Perkin Elmer).

Secondly, a tumor growth study was conducted to determine the optimal timeframe post inoculation for delivering H-FIRE treatment to tumor bearing rodents. Tumor growth was characterized *via* IVIS bioluminescence. Additionally, rodents were sacrificed on days 7, 14, 21, and 28 to acquire T2W MRI (7.0 T), H&E-stained tumor dimensions, and end-point immune cell infiltration.

### 2.4 High-frequency irreversible electroporation

**Figure 1:**
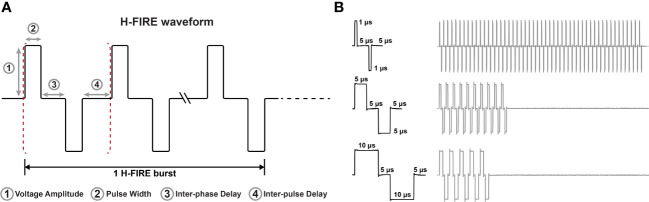
Representative H-FIRE waveforms. **(A)** Schematic that represents the nomenclature associated with the H-FIRE waveform and **(B)** recording of voltage waveforms during treatment depicting differences in pulse width and the burst period.

A custom pulse generator (VoltMed Inc., Blacksburg, Virginia) was used to deliver bursts of biphasic pulsed electric fields both *in vitro* and *in vivo*. This generator is capable of producing voltage waveforms with pulse rise times of 100 ns and a maximum voltage/current output of 5kV/100A. Voltage and current waveforms were recorded using a WaveSurfer 2034z oscilloscope (Teledyne LeCroy, Chestnut Ridge, NY) with a 1000X high voltage probe (Enhancer 3000, BTX, Holliston, MA) and 10X current probe (2877, Pearson Electronics, Palo Alto, CA). Schematics of the three H-FIRE waveforms investigated are shown in **Figure 1** for the 1-5-1 μs, 5-5-5 μs, and 10-5-10 μs waveforms.

### 2.5 Fabrication of collagen hydrogel tissue mimics for *in vitro* H-FIRE studies

**Figure 2:**
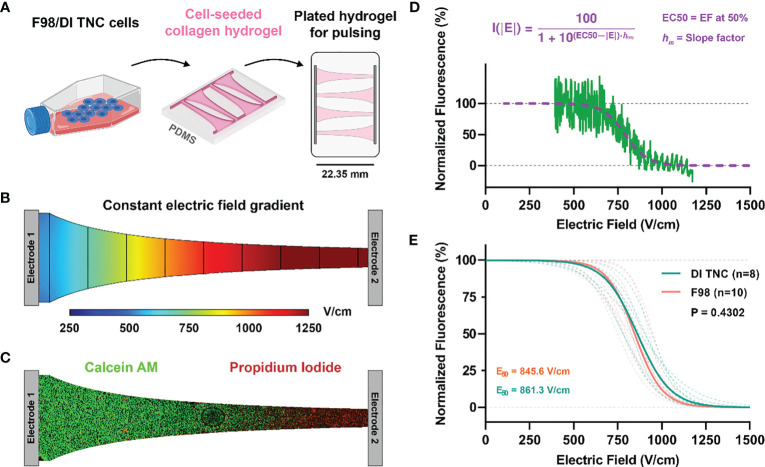
**(A)** Schematic of cell lines (either healthy DI TNC or malignant F98) were individually cultured and then seeded within a microfluidic device experiencing a **(B)** constant electric field gradient as demonstrated by a numerical simulation of the channel. **(C)** Live/dead fluorescent imaging of calcein/PI-stained cells demonstrated a **(D)** sigmoidal fall off of fluorescence along the narrow band of the channel that was then correlated with specific electric field values as determined by numerical methods. **(E)** Electric field thresholds for both DI TNC and F98 cell lines were determined by the EC50 correlating with the 50% survival point.

Type I collagen from rat tail (Corning) was neutralized using 1N NaOH solution (Sigma) (2% of initial collagen volume) followed by dilution to a final concentration of 5 mg/ml using 10X DMEM (Sigma) (10% of the final volume) and 1X DMEM (ATCC). Cells, either healthy rodent astrocytes (DI TNC1) or rodent glioma (F98), suspended in solution at a density of 2×10^6^ cells per milliliter. PDMS (Polydimethylsiloxane) molds were used to form the collagen hydrogel scaffolds into the geometry shown in **Figure 2**. To enhance the attachment of collagen to PDMS, the molds were pretreated with 1% PEI solution (Acros organics) for 10 minutes followed by a 20-minute treatment with 0.1% glutaraldehyde (Fisher chemical) solution. Approximately 400 μl of collagen mixture was placed in each PDMS mold (100 μl in each chip × 4). PDMS molds were placed into a rectangular 8 well plate, followed by 25 minutes of incubation at 37°C. 2 μl of media was added on top of the scaffolds after solidification and hydrogels were incubated for 24 hours prior to pulsing.

Treatment for *in vitro* tumor constructs was accomplished using a mobile incubator maintained at 37°C. A sample size n > 8 was achieved for each H-FIRE waveform and each cell line. After treatment, media was replenished and cells were incubated for another 24 hours to allow for development of the lesion. For staining, media was aspirated from each well and replaced with aa staining solution consisting of PBS, 2 μM calcein AM (Invitrogen), and 23 μM PI (Invitrogen). Hydrogels were incubated at 37°C and 5% CO2 for 30 minutes, thereafter, were washed twice with PBS prior to imaging using an inverted microscope (DMI 6000B, Leica Microsystems) with a 5× objective. The appropriate filters were used to image calcein AM (Ex:460–500; DC:505; EM:570–640) and propidium iodide (EX:545/26, DC:565, EM:605/70).

Images were processed in Image J (National Institute of Health (NIH)). A rectangular (22.35 × 1.35 mm) region of interest (ROI) was defined which spans the length of the chip, the intensity of each channel was averaged across the width of the ROI. This analysis yielded a profile describing the average intensity of calcein/PI as a function of distance along individual chips. Numerical methods (described below) were then used to solve for the electric field distribution along this chip, and the relationship between the local electric field and the distance along the chip was extracted. Finally, the datasets were mapped to one another, and the average intensities were normalized to then obtain the normalized intensity vs. local electric field (**Figure 2**).

### 2.6 Numerical methods for determination of pulsing parameters

A numerical model was constructed in COMSOL Multiphysics v5.6 (COMSOL Inc., Stockholm, Sweden) to determine the electrode configuration and pulsing parameters for adequate tumor coverage in ablative electric fields. The tumor was approximated as a 12.6 mm^3^ sphere, as determined by the tumor growth study, and a realistic rodent brain domain was defined using a 3D reconstruction from a T1W MRI scan (3D Slicer 4.11). The final domain, including the brain, tumor sphere, and two monopolar electrodes, consisted of 207,836 tetrahedral elements resulting from an “extra fine” mesh setting within COMSOL. After mesh generation, the electric potential distribution was modeled using Equation 1.


(1) ∇· (σ∇ϕ)=0

Here, *σ* is the electrical conductivity as a function of the electric field E and *ϕ* is the electric potential.

A pre-treatment voltage ramp was performed to characterize the baseline electrical conductivity reflecting the tissue properties of tumor tissue. Results from the *in vitro* study informed the use of the H-FIRE 5-5-5 μs burst scheme to be applied *in vivo* (Section 3.1). Thus, for treatment of glioma-bearing rodents, H-FIRE therapy was administered as 200 bursts (100 µs of energy per burst) delivered at a rate of 1 burst per second, and a delivery across two blunt-tipped stainless steel electrodes. Parameters including applied voltage, electrode exposure, and electrode spacing were parametrically swept through until desired field distributions were predicted. Two desired protocols were established based on the field distribution: the high-dose H-FIRE protocol, where strong ablation-strength fields predominantly encapsulated the tumor, and the low-dose H-FIRE protocol, where low-strength BBB disruption strength fields encapsulated the majority of the tumor while minimizing the extent of ablation strength fields. The latter was utilized to observe the effects of enhanced delivery of a combinatorial adjuvant from the expected BBB disruption while eliminating the therapeutic effects of ablative strength high-dose H-FIRE fields.

Volume coverage was based on field thresholds from *in-vitro* studies (861.3 V/cm for ablation coverage) and previous *in-vivo* work from our group (113.5 V/cm for BBB disruption volume coverage (17)). Numerical modeling informed use of a 2.5 mm electrode exposure, 3 mm electrode spacing, and an applied potential of 180 V or 525 V producing a voltage-to-distance ratio of either 600 V/cm (low dose BBB disruption protocol), or 1,750 V/cm (high-dose ablation protocol) respectively. On treatment day, the electrodes were advanced into the brain using the micromanipulator arm of the stereotactic frame according to stereotactic coordinates references to the location of the rostral electrode (bregma 4mm caudal, 3.5 mm lateral, at a depth of -4 mm relative the surface of the dura).

**Figure 3:**
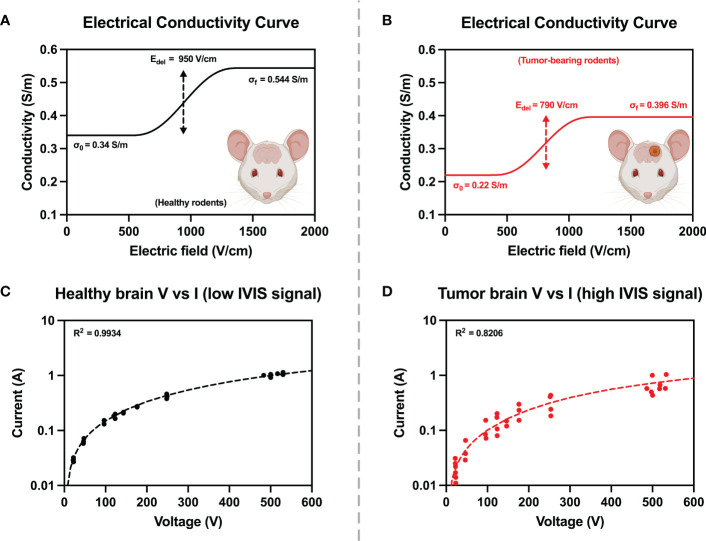
Electrical tissue conductivity curves for **(A)** healthy and **(B)** tumor-bearing rodents were reconstructed by ensuring agreement between experimental and numerical values from pre-treatment voltage and current ramps **(C, D)**. Numerical modelling was used to manipulate parameters of the conductivity curves until simulated current and voltage (dashed lines) were in good agreement with experimentally measured values (solid points).


Three bursts of 25, 50, 100, 150, 180 (low-dose treatment voltage), and 525 V (high-dose treatment voltage) were applied. The low-dose and high-dose H-FIRE groups received voltage ramps only up to their respective treatment voltage. Corresponding voltage and current profiles were recorded on an oscilloscope (Teledyne LeCroy, Chestnut Ridge, NY, USA) with a 1000× high voltage probe (Enhancer 3000, BTX, Holliston, MA, USA) and 10× current probe (2877, Pearson Electronics, Palo Alto, CA, USA). The final 2 μs of the final pulse for each recorded voltage and current burst was averaged and plotted on a current vs voltage plot. Experimental conditions were replicated in COMSOL Multiphysics v5.6 (COMSOL Inc., Stockholm, Sweden) and initial electrical properties were derived from reported values for healthy rodent astrocyte tissue (17) and malignant canine glioma tissue (26). A comprehensive parametric study varying parameters σ_0_, E_del_, E_range_, and A was performed to design two sigmoidal conductivity curves, fitting the experimental voltage and current data collected from the pre-treatment voltage ramps for healthy rodent brain, and malignant tumor tissue (**Figure 3**). Healthy brain tissue properties were determined in rodents with low or no IVIS signal. The conductivity sigmoid is comprised of an initial value σ_0_ which represents the initial conductivity of the tissue prior to entering an electroporated state, and plateaus to a value of σ_f_ once the tissue has entered a state of complete electroporation. The transition of an un-electroporated to electroporated state occurs over a range ± 2·E_range_ centered about a point E_delta_.

(2) $$\sigma \left(E\right)=\;\sigma_{0}\;\left(1+A\;\cdot \;flc2hs\left(E-E_{del},E_{range}\right)\right)$$

Following the characterization of rat brain tissue conductivity, an electric potential boundary condition (*ϕ* = 180V or 525V) and a grounding boundary condition was applied on either electrode. Thermal dissipation and Joule heating effects were calculated using a modified bioheat equation (Equation 3):


(3) $$\rho c_{p}\frac{\partial T}{\partial t}=\nabla \cdot \left(k\nabla T\right)+\omega_{b}\rho_{b}c_{b}\left(T_{b}-T\right)+\frac{\sigma \cdot \;{\left|E\right|}^{2}\cdot p}{\tau }$$

where *ρ* is the tissue density; *c_p_* the specific heat; *k* is the thermal conductivity; and *ω_b_* is the distributed blood perfusion coefficient. In this study, *T_b_*, *ρ_b_*, and *c_b_* were 32.2°C, 1050 kg/m^3^, and 3617 J/(kg·K), respectively. The terms *p* and *τ* represent the duty cycle normalization terms, which allow for high frequency electroporation thermal contributions to be represented as a continuous heat source rather than a periodic heat source, as the latter would require drastic changes in solver time-stepping. Here *p* is the burst on-time (100x10^-6^ s) and *τ* is the period of burst delivery (1 s). Additional parameter values used in this model are represented in **Table 1**.

**Table 1:** Electrical and thermal values for the numerical model.

Material	Parameter	Value	Units
Brain tissue	Density, ρ	1046	kg/m^3^
	Specific heat, c	3630	J/(kg·K)
	Thermal conductivity, k	0.51	W/(m·K)
	Blood perfusion coefficient, ω	1.75x10^-3^	1/s
Insulation	Density, ρ	1190	kg/m^3^
	Specific heat, c	1470	J/(kg·K)
	Thermal conductivity, k	0.18	W/(m·K)
	Electrical conductivity, σ	2.5x10^-14^	S/m
Stainless steel	Density, ρ	7850	kg/m^3^
	Specific heat, c	475	J/(kg·K)
	Thermal conductivity, k	44.5	W/(m·K)
	Electrical conductivity, σ	4.03x10^6^	S/m

Material properties were attained from IT’IS database (https://itis.swiss).

### 2.7 Combinatorial doxorubicin + H-FIRE

To investigate the combinatorial effects of H-FIRE in conjunction with a molecular adjuvant, select rodents were enrolled to receive liposomal doxorubicin either as a monotherapy or as an adjuvant to H-FIRE. The selection of liposomal doxorubicin (Lipodox) as the drug for this model was based on the high expression levels of alkyltransferase in F98 cells. Alkyltransferase is an enzyme that inactivates alkylating agents, such as the standard of care therapeutic agent, temozolomide. This selection was based on a rationale that considers the specific characteristics of F98 cells and the pharmacological properties of Lipodox. These rodents received an IP injection of 5mg/kg Lipodox (2mg/mL, Cardinal Health). According to the manufacturer’s instructions, Lipodox was prepared to a final concentration of 0.5mg/mL in D5W and supplied using a closed system transfer device (Equashield) coupled to a 22G retractable butterfly catheter. To ensure that the complete volume of chemotherapy was delivered, the system was flushed with 0.5 mL of sterile saline. For rodents in the combinatorial high-dose/low-dose H-FIRE + DOX group, Lipodox was administered 30-60 minutes before H-FIRE administration.

Parallel to the survival study, a separate investigation using tumor bearing rodents was used to quantify the concentrations of Lipodox in both tumor and healthy brain tissue. Concentrations were measured in 4 groups (n=3 each): 1) within the homologous contralateral striatum of tumor bearing rat brains of Lipodox treated animals; 2) within the homologous contralateral striatum of tumor bearing Lipodox + H-FIRE treated animals; 3) within the F98 tumor after Lipodox treatment only; and 4) within the F98 tumor after Lipodox and high-dose H-FIRE ablative treatment. One hour after IP liposomal doxorubicin (Lipodox) administration and H-FIRE treatment, rats were sacrificed, and their brains extracted and loaded into a 2 mm matrix slicer brain matrix (Zivic Instruments, Pittsburg, PA, USA). As Lipodox has a plasma half-life of 2–3 days, we presumed the entire representative dose was still present in the circulation at the one-hour time point (34). Transverse sections of the brain containing implanted tumor and a homologous section of unaffected contralateral brain were harvested. Tumor samples were obtained at the peripheral margin of the tumor at its interface with peritumoral brain tissue, as these are contrast-enhancing tumors regions on MRI examinations. Harvested brain sections were trimmed to 0.03 g, placed into 1.5 ml centrifuge tube containing 600 µL of acidified alcohol extraction solution (0.3 N HCl in 50% EtOH), homogenized (TissueRuptor II, Qiagen), and then refrigerated at 4°C for 24 hours. Samples were then centrifuged at 4,000 g for 20 min at 4°C. The supernatant was extracted and intensity measurements (excitation/emission wavelengths: 480/590 nm) performed using a fluorometer (VersaFluor; Bio-Red Laboratories, Hercules, CA). Lipodox concentrations were quantified by linear regression and a standard curve obtained from eight serial stock Lipodox concentrations. The concentration of doxorubicin in each sample was obtained by taking the average of three fluorometric readings. Fluorometric readings were normalized to measurements from normal control animals that only received vehicle (D5W) control treatments (n=3) to correct for autofluorescence.

### 2.8 Survival endpoint determination

Subjects were euthanized once they met the criteria for a humane endpoint as indicated by rodent neurologic severity score (RNSS), physical observation score (PO), body condition score (BC), and body weight (BW). The study was concluded at a timepoint 40 days post treatment. Specific scoring criteria may be found in the **Supplemental Material (Data Sheet 1)**.

### 2.9 Characterizing immune response following H-FIRE treatment

#### 2.9.1 Characterizing the innate and adaptive immune response following H-FIRE treatment

A secondary controlled study was designed to capture the innate and adaptive immune responses following high-dose H-FIRE treatment. In this parallel study, rats were sacrificed at a predetermined endpoint which was either 24 hours (n=4) or 10 days (n=4) post-H-FIRE treatment for innate and adaptive responses, respectively. The euthanasia process involved administering an IP injection of pentobarbital sodium (Fatal-Plus, Vortech Pharmaceuticals) after isoflurane anesthesia. The brains were transversely sectioned into 1mm-thick segments and divided, along with cervical lymph nodes, for preservation and subsequent analysis using either 10% Formalin or RNA later.

##### 2.9.1.1 Serum cytokine analysis

Blood samples were collected through cardiac puncture immediately after euthanasia for serum cytokine analysis. Serum samples from rats with equal survival times were tested using the MAP Rat Cytokine Immunology Multiplex Assay (RECYMAG65K27PMX, Millipore Sigma), allowing for the simultaneous measurement of 14 chemokines and cytokines.

##### 2.9.1.2 Gene expression and pathway analysis

After H-FIRE treatment, the Qiagen RNeasy Lipid Tissue Mini Kit was used to extract total RNA from brain sections. The RNA was quantified and utilized for cDNA synthesis, which was carried out using an RT2 First Strand Kit (Qiagen). The RT2 Profiler PCR Array for Innate and Adaptive Immunity (Qiagen) was utilized for gene expression analysis, and triplicate PCR reactions were performed on each sample. The gene expression data were normalized to internal housekeeping genes and analyzed with online software from the manufacturer, Gene Globe (Qiagen) and iPathways (Advaita). The fold-change in gene expression was calculated using the mean Ct value of triplicate PCR reactions in comparison to the sham controls.

#### 2.9.2 Immunohistochemical analysis

The local immune response to treatment was assessed by characterizing immune cell infiltrates within brain sections collected from high-dose H-FIRE ablation groups at the study endpoint. Tissue samples were sectioned and stained for presence of T cells (CD3, Dako), helper T cells (CD4, Origene), cytotoxic T cells (CD8, Invitrogene), regulatory T cells (FoxP3, Invitrigene), B cells (CD79a, Santa Cruz), microglia (IBA-1, FUJIFILM), M1 macrophages (CD86, Abcam), and M2 macrophages (CD163, Abcam). Methods were followed as presented by Koshkaki et al. (35). Samples were categorized as peritumoral region (healthy tissue), transition zone (submillimeter zone between the tumor mass and the peritumoral region), tumor mass, and the necrotic core. Tissue samples were imaged using a Nikon Eclipse Ni-U microscope using a Ds-Ri2 camera. The acquired images were analyzed using the NIS element BR software version 5.21.01.

Briefly, auto thresholding was done on all images followed by greyscale conversion. Mean gray values (*MGVi*) and gray area fractions (*AF*) were calculated using the software. The values were then used to find the final chromogen intensity (*f*) as follows:


(4) f=255−MGVi

Mean *f* and *AF* values were next calculated using five high power fields from each slide. The immunohistochemistry (*IHC)* score was calculated using the mean values and the following equation:


(5) IHC=f·AF

### 2.10 Statistical analysis

GraphPad Prism v9.4 (GraphPad Software, San Diego, CA) was used to conduct statistical analysis in all cases. A Mantel-Cox Logrank test was used to evaluate differences among survival curves with respect to the sham control group. Changes between individual treatment groups and sham controls for gene expression, cytokine levels, and IHC scores were evaluated *via* Student’s t-test. Multiple comparisons between treatment groups were conducted using a two-way analysis of variance (ANOVA) followed by a Tukey’s *post hoc* test where the criteria for significance was set to alpha = 0.05 (p< 0.05).

## 3 Results

### 3.1 Determination of H-FIRE pulsing parameters in an *in vitro* collagen hydrogel tumor scaffold

An H-FIRE waveform is constructed as: positive phase – inter-phase delay – negative phase – inter-pulse delay, with this burst of bipolar pulsed electric fields is repeated until a desired energized-time (100 μs) is reached. Cell death from H-FIRE therapy was investigated for three distinct waveforms: 1) 10-5-10 μs, 2) 5-5-5 μs, 3) 1-5-1 μs. A 3D collagen hydrogel platform was utilized for viability experiments. These hydrogels allow cells to adhere to the collagen matrix and express physiologically relevant morphologies, facilitating characterization of electroporation *in vitro*. H-FIRE therapy was administered, and cell viability was assessed using calcein green and propidium iodide 24 hours after pulsing.

The geometry of the hydrogel was formed such that a linearly varying (constant gradient) electric field is induced across the cells (**Figures 2A–C**). Rather than quantifying a discrete electric field which elicits cell death, we can describe a sigmoidal relationship between the applied electric field and percent cell death. Numerical methods were used to solve for the electric field distribution along this chip, this was subsequently mapped to the normalized intensity to attain viability plots (**Figures 2D, E**). Nonlinear regression was conducted to fit the normalized intensity vs. electric field data sets to a sigmoid equation (Equation 6). Here, *I(|E|)* describes the normalized intensity (*I*) at a specific electric field magnitude (*|E|*), *EC50* is the electric field corresponding to 50% viability, and *h_m_* is the slope of the transition.


(6) $$I\left(\left|E\right|\right)\;=\frac{100}{1+10^{\left(EC50-\left|E\right|\right)\cdot h_{m}}}$$

The fitting parameters of Equation 6 are highlighted in **Table 2**. Direct comparison of the viability plots demonstrates that longer pulse widths elicit cell death at lower electric fields for both the F98 and DI TNC1 cell lines. Translating the results for *in vivo* ablation, these results suggest that for given voltage and pulsing parameters, a larger volume of cell death is induced with the 10-5-10 μs waveform in comparison to the others. Interestingly, there is a subtle separation of induced cell death between the healthy DI TNC1 cell line and the malignant F98 cell line using the higher frequency waveform 1-5-1 μs. This result implies H-FIRE can be administered in such a way that cell death is achieved in malignant cells while sparing, to a limited extent, underlying healthy astrocytes.

**Table 2:** F98 and DI TNC cell viability parameters for H-FIRE in collagen hydrogel scaffolds.

Pulse Waveform	Cell line	50% viability, EC50	Slope, h_m_
1-5-1 μs	F98 (n = 9) DI TNC1 (n = 11)	961.1 ± 46.0 V/cm 1255.5 ± 122.7 V/cm	-6.03 × 10^3^ ± 1.91 × 10^3^ -3.83 × 10^3^ ± 1.04 × 10^3^
5-5-5 μs	F98 (n = 10) DI TNC1 (n = 8)	845.6 ± 63.3 V/cm 861.3 ± 59.9 V/cm	-5.55 × 10^3^ ± 1.35 × 10^3^ -4.61 × 10^3^ ± 0.97 × 10^3^
10-5-10 μs	F98 (n = 8) DI TNC1 (n = 9)	643.0 ± 22.6 V/cm 667.0 ± 20.4 V/cm	-7.53 × 10^3^ ± 0.89 × 10^3^ -5.95 × 10^3^ ± 1.52 × 10^3^

For the DI TNC1 cell line, the slope of the sigmoid, h_m_, indicates large pulse widths incur cell death more efficiently, as |h_10-5-10_| > |h_5-5-5_| > |h_1-5-1_|. Ultimately, a balance between the extent of cell death and anticipated nerve excitation was desired. The effects of pulse width on nerve excitation were previously studied demonstrating greater excitation at lower frequency waveforms (36).

Numerical modeling for *in vivo* treatments indicated that the high-dose H-FIRE protocol was estimated to produce 98.7% and 100% coverage of the tumor in ablation strength and BBB disruption strength fields respectively while the low-dose H-FIRE protocol was predicted to produce 6.7% and 100% ablation strength and BBB disruption strength fields respectively (Figure shown in **Supplemental Material (Data Sheet 1)**.

### 3.2 Validation of Lipodox perfusion into the brain following H-FIRE

**Figure 4:**
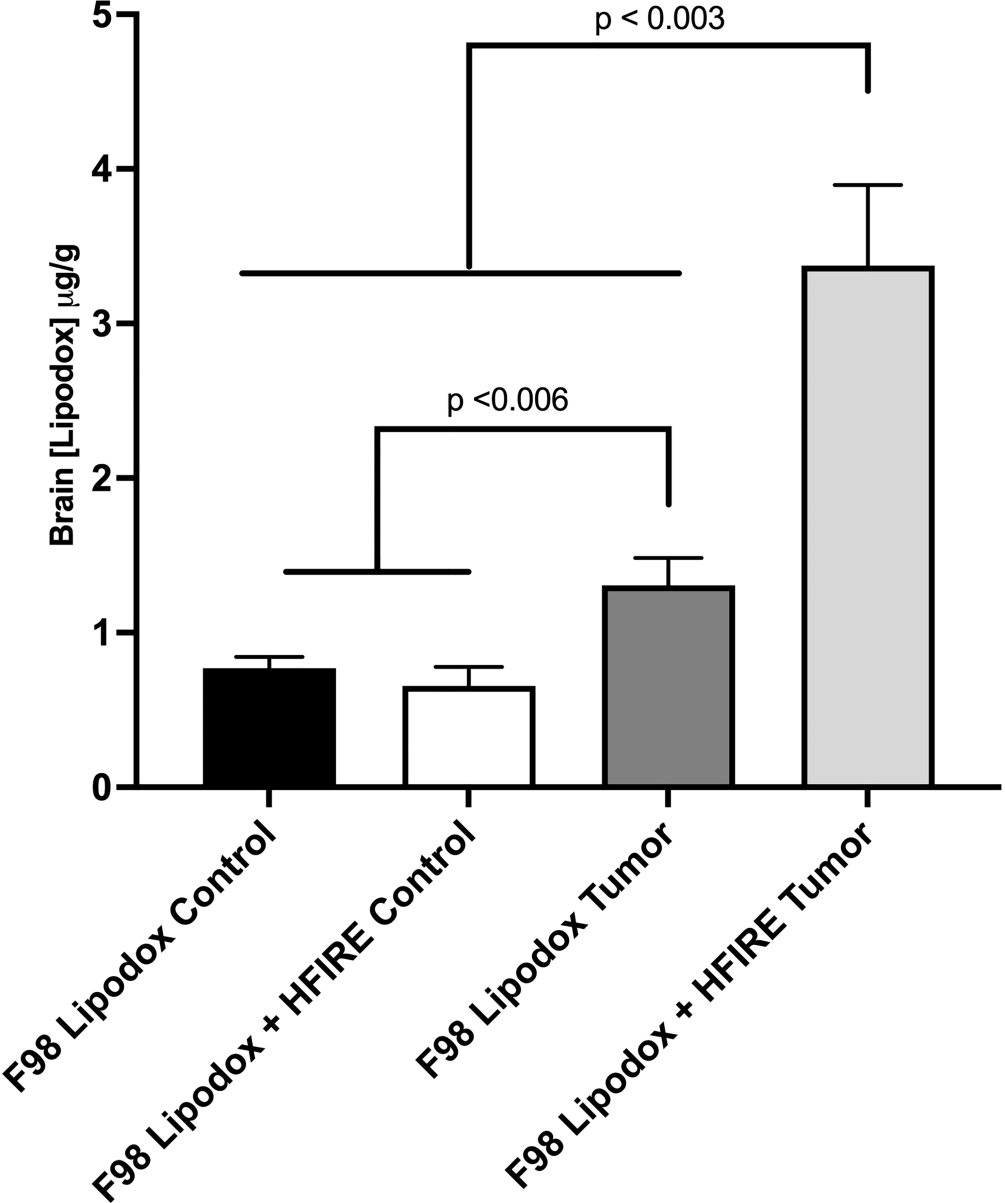
Concentrations of Lipodox in bran tissue were measured in four groups (n=3 each) one hour after intraperitoneal injection. All groups received Lipodox injection. The groups include 1) F98 Lipodox Control in which Lipodox concentrations were measured in the normal striatum of the brain contralateral to the tumor, 2) F98 Lipodox + HFIRE Control in which Lipodox concentrations were measured in the homologous, normal striatum of the brain contralateral to the tumor, 3) F98 Lipodox Tumor in which concentrations were measured within the tumor, and 4) F98 Lipodox + HFIRE Tumor in which concentrations were measured within the tumor. Although a detectable difference was noted between the concentrations of Lipodox measured within the healthy part of the brain and within the tumor, H-FIRE treated rodents had a much more significantly elevated concentration in Lipodox concentrations in the tumor. H-FIRE did not increase the concentration of Lipodox in the healthy striatum compared to that of the Lipodox Control.

Lipodox concentrations are reported in **Figure 4**. The findings of the study showed that while significantly higher concentrations of Lipodox were still detected in the Lipodox only F98 group (p< 0.006), the use of high-dose H-FIRE significantly improved the penetration of Lipodox into the brain tissue compared to all other groups (p< 0.003).

### 3.3 Characterization of the bioluminescence kinetic curve and tumor growth study

**Figure 5:**
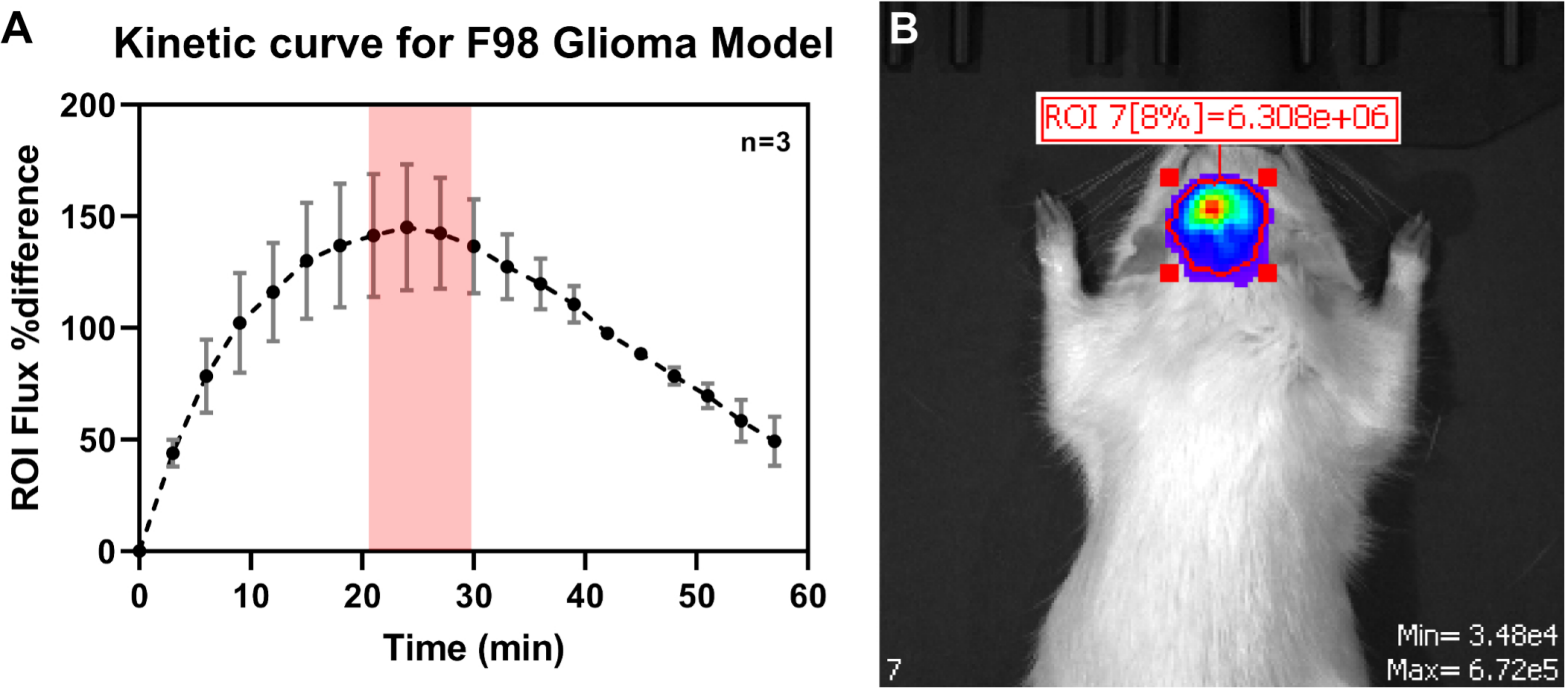
**(A)** Determination of the kinetic curve for quantification of tumor bioluminescent signal during IVIS imaging. The maximum luminescent signal reading was found to be 20-30 miutes following IP administration of D-Luciferin. **(B)** Representative bioluminescent image during the tumor growth kinetic study.

First, the kinetics of bioluminescence was quantified, as both the tumor type and tumor implant location are known to affect the bioluminescent kinetics (**Figure 5B**). The maximum intensity signal was found to be between 20-30 minutes (n=3) following intraperitoneal administration of D-luciferin (**Figure 5A**).

### 3.4 *In vivo* tumor growth study

**Figure 6:**
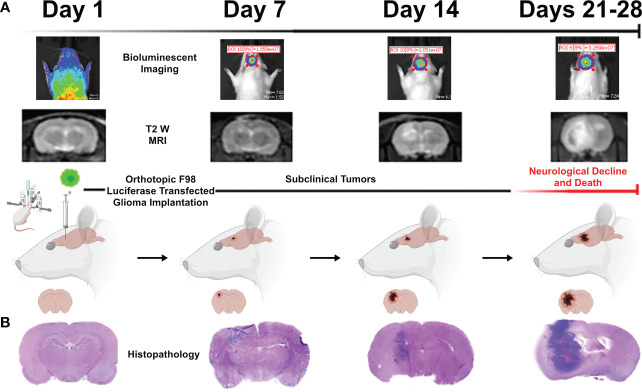
Representative images acquired during the tumor growth study *via* **(A)** bioluminescent IVIS imaging, T2W MRI, and **(B)** representative brain histopathology. These three methods were used to quantify tumor growth of untreated rodents over the course of a four-week period to determine the optimal period for treatment intervention (found to be 7 days). Figure created with https://BioRender.com.

We used IVIS bioluminescence, MRI, and H&E (**Figures 6A, B**) to characterize tumor growth. Day 7 was found to be an optimal time point for treating rodents, as the tumor grew large enough for visualization without surpassing the two-electrode treatment zone or inducing severe neurological decline or death.

### 3.5 Survival endpoint study

**Figure 7:**
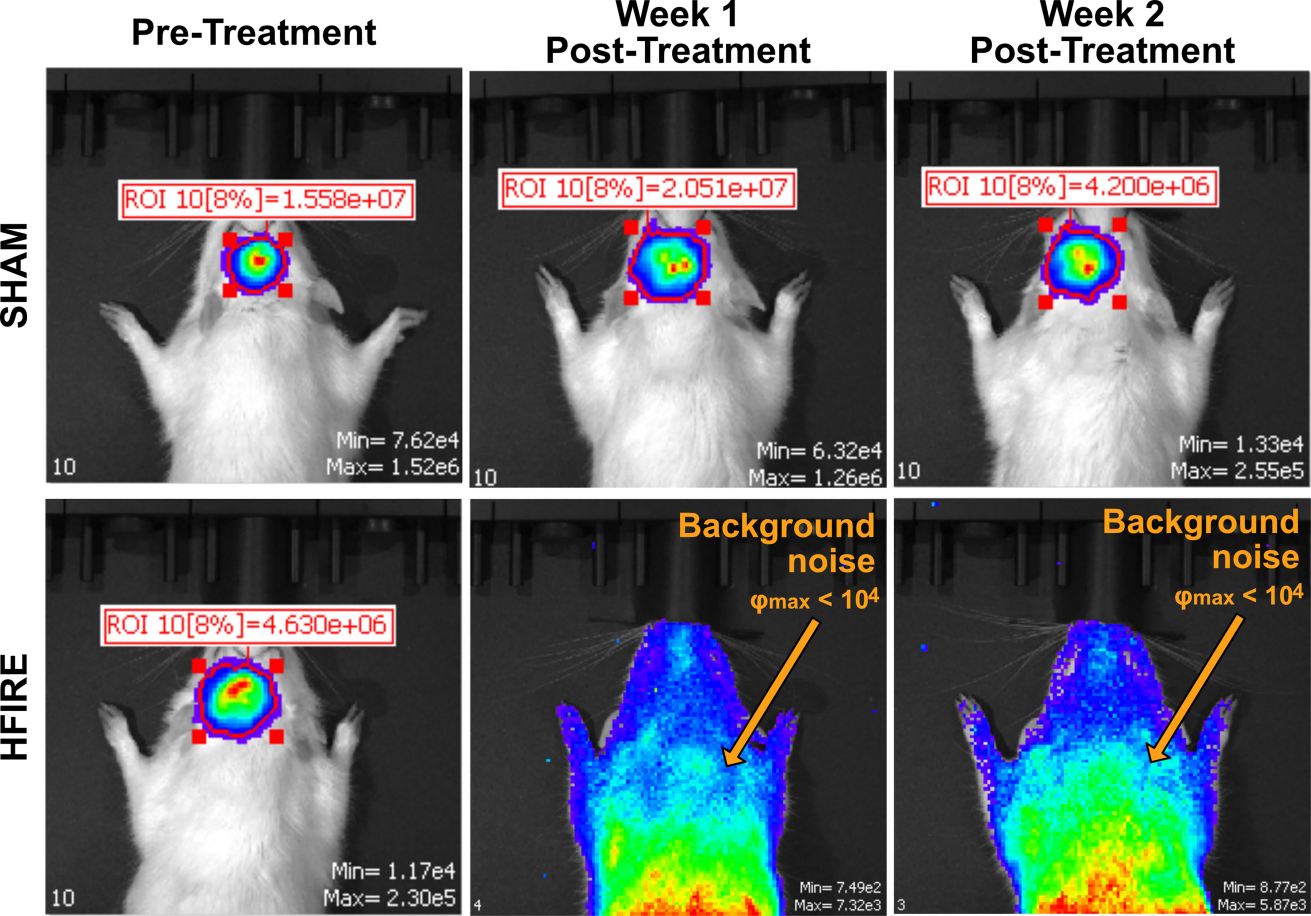
Bioluminescent imaging from the IVIS imaging system was used to monitor tumor growth throughout the duration of the study. A representative rodent from the Sham group is shown on the top row while a representative rodent from the high dose H-FIRE therapy group is shown on the bottom row. The individual rodents were imaged at time points 7 days post tumor implantation (pre-treatment), and at time points one and two weeks post treatment. The rodent from the Sham group which, received tumor implantation but no H-FIRE therapy, demonstrates strong presence of the tumor signal throughout the three week period shown, while the tumor bearing rodent receiving H-FIRE therapy demonstrates eradication of the tumor signal one week post treatment. Maximum fluence values under 10^4^ were considered background noise and not strong enough to be considered a tumor signal.

Animals underwent IVIS imaging weekly to monitor progression of tumor growth. While all rodents were inoculated with tumors, the SHAM group did not receive any therapeutic intervention. Representative images of the IVIS signals for SHAM and H-FIRE treated rodents are shown in **Figure 7**. The SHAM group demonstrated a strong tumor signal throughout the duration of the monitoring period, whereas H-FIRE treated rodents that demonstrated strong tumor signals prior to receiving treatment, demonstrated no relevant signal during follow up imaging, suggesting eradication of the tumor. Photon fluence (φ) with a maximum signal measuring less than 10^4^ was considered to be background noise.

Body weight, physical observation (PO) scores, and rodent neurological severity score (RNSS) scores were recorded weekly to monitor potential signs of disease progression. Overall, the animals body weight followed rising trends over the course of the study (data not shown).

**Figure 8:**
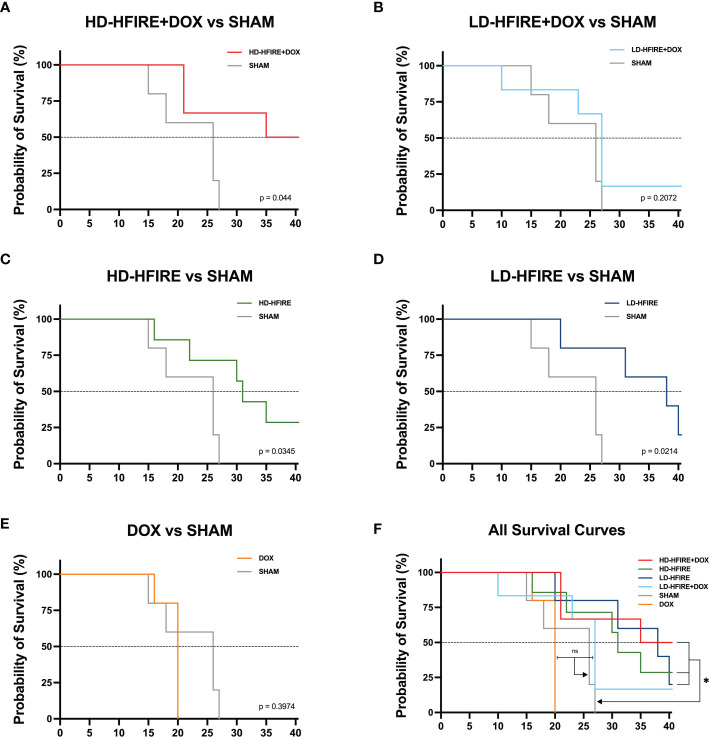
Kaplan-Meier survival plots for F98 tumor bearing rats. The **(A)** high-dose H-FIRE+Lipodox (HD-HFIRE+DOX), **(C)** high-dose H-FIRE (HD-HFIRE), and **(D)** low-dose H-FIRE (LD-HFIRE) lived significantly longer than the sham controls (p< 0.05). Significance was not noted between the **(B)** low-dose H-FIRE +Lipodox (LD-H-FIRE+DOX) or **(E)** Lipodox only (DOX) groups against the sham. **(F)** Overall, H-FIRE intervention appears to significantly improve overall rodent survival (with the exception of the LD-H-FIRE+DOX group). ns = not significant; *p < 0.05.

The survival curve (**Figure 8**) demonstrates that H-FIRE significantly lengthened the lives of animals with the F98 glioma tumor (with the exception of the low-dose H-FIRE + DOX group). The groups were each compared for compared survival against the sham group, and overall survival fractions were as follows: 50% for high-dose H-FIRE+DOX (p = 0.044), 28.6% for high-dose H-FIRE (p = 0.034), 20% for low-dose H-FIRE (p = 0.0214), 17% for low-dose H-FIRE+DOX (ns; p = 0.2072), and 0% for the DOX group (ns; p = 0.2974). Statistically greater survival compared to the sham group was seen in high-dose H-FIRE+DOX (p = 0.044), high-dose H-FIRE (p = 0.034), and low dose H-FIRE (p = 0.0214). Low-dose H-FIRE+DOX (ns; p = 0.2072) and the DOX only group (ns; p = 0.2974) did not show any improvements in survival outcomes. On day 26 post-treatment, all five rats in the Sham group met humane endpoint while 3/6 (50%) high-dose H-FIRE+DOX, 2/7 (29%) high-dose H-FIRE, 1/5 (20%) low-dose H-FIRE, and 1/6 (17%) low-dose H-FIRE+DOX treated rats survived to the conclusion of the trial (day 40).

### 3.6 Monitoring the Immune response after H-FIRE treatment

**Figure 9:**
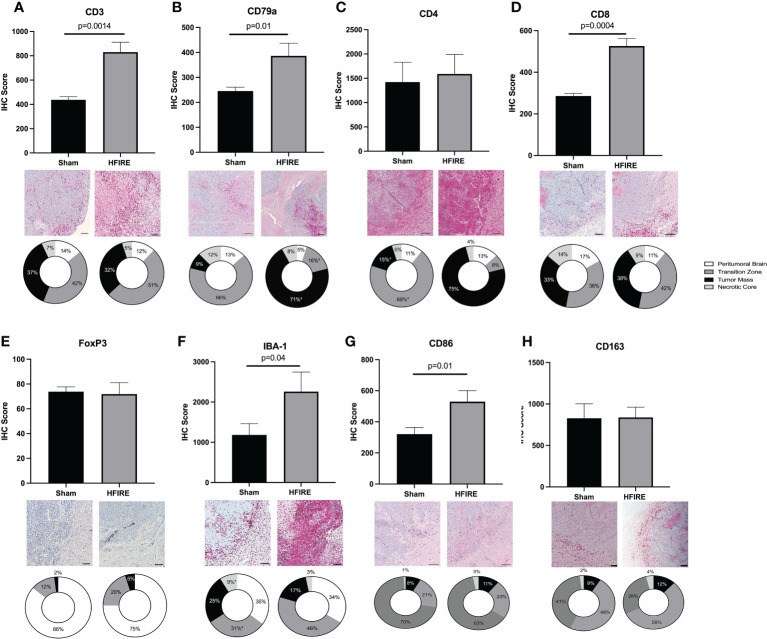
Immune cell infiltration after H-FIRE ablation. Brain tissue sections were stained for **(A)** T cells (CD3), **(B)** B cells (CD79a), **(C)** helper T cells (CD4), **(D)** cytotoxic T cells (CD8), **(E)** regulatory T cells (FoxP3), **(F)** macrophages/microglia (Iba-1), **(G)** M1 macrophages (CD86), and **(H)** M2 macrophages (CD163). The proportion of each cell type was studied with regards to proximity to the tumor mass. Immune cell infiltrates were analyzed within the following regions: peritumoral brain, transition zone, tumor mass, and necrotic core. Bar = 150 μm in all panels.

Local immune cell infiltration was investigated *via* IHC staining of brain tissue samples collected at the study endpoint (**Figure 9**). The overall presence of T cells, B cells, and microglia was observed to be greater in the H-FIRE ablation group compared to the sham controls. Furthermore, CD8 cytotoxic T cell infiltration was found to be higher in the H-FIRE ablation group, while the density of CD4 helper T cells and immunosuppressive regulatory T-cells was similar between H-FIRE ablation and sham control groups.

When comparing immune cell infiltrates between the previously defined treatment zones (Section 2.9.2), the proportion of B cells was much higher within the tumor mass (71% in the H-FIRE ablation group vs. 9% in the sham control group), while the proportion of T cells and microglia was higher within the transition zone. Interestingly, when differentiating T cell subtypes, we observed a greater proportion of CD4 helper T cells within the tumor mass (75% in the H-FIRE ablation group vs. 15% in the sham control group), whereas the proportion of cytotoxic T cells and regulatory T cells was greatest within the transition zone of the H-FIRE ablation group.

**Figure 10:**
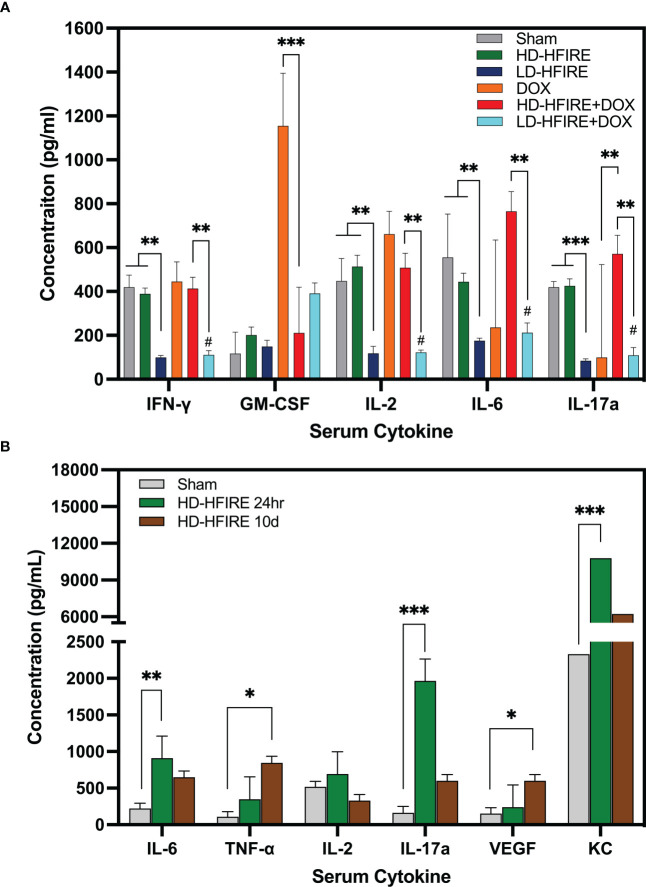
**(A)** Summary of cytokine concentrations across the five treatment groups. Compared to sham-treated controls, IFNγ, IL-2, IL-6, and IL-17a serum cytokine concentrations were significantly decreased following low-dose (BBB disruption) H-FIRE. IFNγ, IL-2, IL-6 and IL-17a were significantly increased following high-dose (ablation) H-FIRE treatment relative to the low-dose (BBB disruption) H-FIRE treatment group. GM-CSF was significantly decreased following treatment with high-dose (ablation) H-FIRE + Lipodox relative to Lipodox alone, whereas IL-17a was significantly increased. **(B)** In a separate group of rodents sacrificed at 24 hours or 10 days post-H-FIRE treatment, IL-6, IL-17a and KC were significantly increased at 24h post-H-FIRE ablation relative to the sham controls, whereas TNF-α and VEGF were significantly increased at 10d post- H-FIRE ablation. (*p< 0.05, **p< 0.01, ***p< 0.001, # group significantly different from sham p<0.01).

The present study conducted a comparison of serum cytokine concentrations in rats treated with H-FIRE ablation and H-FIRE BBB disruption. Fourteen cytokines were analyzed, and the results showed that the levels of interferon-gamma (IFNγ) (p< 0.01), interleukin-2 (IL-2) (p< 0.01), interleukin-6 (IL-6) (p< 0.01), and interleukin-17a (IL-17a) (p< 0.001) were significantly elevated in rats treated with H-FIRE ablation (**Figure 10A**). This trend was consistent for IFNγ, IL-2, IL-6 and IL-17a even after the administration of Lipodox chemotherapy, where the levels of these cytokines were increased in rats treated with H-FIRE ablation + Lipodox compared to those treated with H-FIRE BBB disruption + Lipodox. In addition, GM-CSF was found to be significantly decreased in rats treated with H-FIRE ablation + Lipodox chemotherapy relative to rats treated with Lipodox alone. Conversely, IL-17a was significantly increased (p< 0.01) in the same group of rats. At 24 hours post-H-FIRE ablation, there was a significant increase in the levels of IL-6 (p< 0.01), IL-17a (p< 0.001) and keratinocyte-derived chemokine (KC) (p< 0.001) compared to the sham-treated controls, as shown in **Figure 10B**. Additionally, tumor necrosis factor-alpha (TNF-α) and vascular endothelial growth factor (VEGF) levels were significantly increased at 10 days post-H-FIRE ablation (p< 0.05). There were no significant differences in cytokine concentrations observed between the 24-hour and 10-day post-H-FIRE ablation groups.

**Figure 11:**
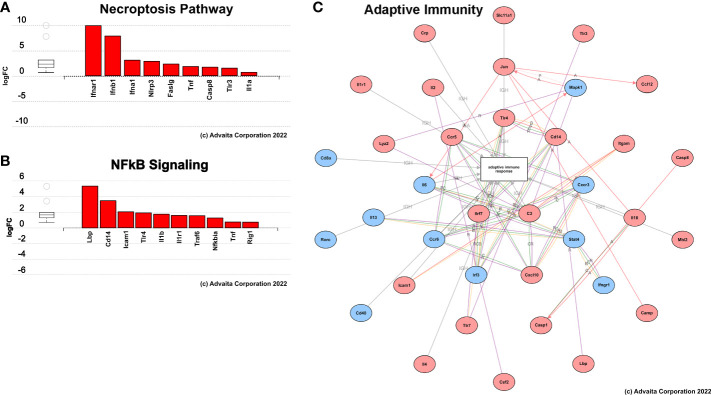
Summary of results from pathway analysis performed using iPathwaysGuide. Genes involved in **(A)** necroptosis, **(B)** NFkB signaling, and **(C)** adaptive immune response pathways were significantly upregulated (red) 10 days post-H-FIRE ablation compared to sham-treated controls.

Gene expression and pathway analysis indicated significant variations in the expression of genes involved with innate and adaptive immunity between treated F98 rat brains and untreated controls (**Figure 11**). At 24-hours post-H-FIRE ablation, most analyzed genes were elevated by more than 2-fold in comparison to sham-treated controls. However, only one gene (*Itgam*) was statistically significant (p< 0.05). At 10-days post-H-FIRE ablation, significant increases in gene expression were observed in H-FIRE ablation groups in comparison to sham-treated controls. These genes included those related to P/DAMP signaling (e.g., *Il6, Tlr1, Tlr4, Il1b, Il18*), necroptotic tumor cell death (e.g., *Casp1, Casp8, Il1b*), and activation of the adaptive immune system (e.g., *Cd80, Cd8a, Ccr5, Cxcl10*). *Casp1, Casp8, Ccl12, Cd8a, Cxcl10, Il2, Lbp, Tlr1, Tlr3*, and *Tlr4* showed the most significant statistical significance (p< 0.01) among the genes upregulated by H-FIRE ablation. In addition, the NF-kappa B (NF-κB) pathway was found to be significantly upregulated relative to sham-controls. NF-κ;B plays an important role in regulating genes associated with inflammation and is responsible for the production of pro-inflammatory cytokines and regulating the development of naive T helper cells into effector T helper cells (Th1, Th2, and Th17 cells), and activated macrophages into the proinflammatory M1 phenotype. The serum cytokine analysis also revealed significant increases in pro-inflammatory cytokines mediated by NF-κ;B following H-FIRE ablation relative to sham controls.

Overall, our results suggest that H-FIRE ablation promotes immune cell infiltration within the tumor microenvironment. Furthermore, these results allude to a systemic immune response induced by H-FIRE treatment of gliomas.

## 4 Discussion

Here we have built upon the preclinical studies of first-generation IRE therapy in the brain and have evaluated the efficacy of both monotherapy and combinatorial (liposomal doxorubicin) H-FIRE for GBM. In summary, a three-dimensional collagen hydrogel tissue scaffold was leveraged to investigate H-FIRE cell death in healthy rodent astrocyte (DI TNC1) and rodent malignant glioma cells (F98). This *in vitro* data served to determine the desired pulsing parameters to be implemented *in vivo*. In parallel to this *in vitro* study, the tumor growth characteristics of F98 cells were quantified in a subset of rodents *in vivo* to inform timing of H-FIRE therapy. Thereafter, the survival endpoint study was initiated in six groups of rodents (**Table 3**) all inoculated with F98 glioma cells. The rodents were monitored for a period up to 40 days post H-FIRE treatment.

**Table 3:** Rodent Cohort Summary.

Name	Tumor (Y/N)	H-FIRE Voltage (V)	Liposomal Doxorubicin (Y/N)	Sample Size (n)	Median Survival (Days)	Overall Survival (%)
Sham	Y	N/A	N	5	26	0
DOX	Y	N/A	Y	5	20	0
Low-dose H-FIRE	Y	180	N	5	38	20
High-dose H-FIRE	Y	525	N	7	31	28.6
Low-dose H-FIRE + DOX	Y	180	Y	6	27	16.7
High-dose H-FIRE + DOX	Y	525	Y	6	37.5	50

As improved overall survival and median survival were seen in all rodents whose treatment protocol included H-FIRE therapy when compared against the sham group, our findings suggest that H-FIRE therapy in the brain extends overall survival time. Meanwhile, the reduction of survival in the DOX only group may indicate that the drug alone induced more toxicity than positive effects. Given that the BBB was not disrupted with this protocol, Lipodox was likely left to systemically circulate, primarily targeting healthy anatomy while the tumor was left to grow. The results from **Figure 4** suggest that H-FIRE treatment leads to a higher global concentration of Lipodox in tumors thus, improving its efficacy. However, the authors note that the low-dose H-FIRE protocol was not tested alongside the high-dose protocol in the concentration study. While we note that LD-HFIRE + DOX was not worse than the sham control, there was no statistical improvement—suggesting that the systemic toxicity induced by the Lipodox may have outweighed the benefit of the concentration that was able to penetrate into the brain with the low-dose H-FIRE protocol. From this, we may infer that sufficient BBB disruption was not achieved with this low-dose protocol. The authors recommend that for future investigations, a more comprehensive study of drug penetration into the brain be conducted to confirm that both protocols are adequate for achieving effective drug perfusion. This recommendation is based on the results from **Figure 4** which show that the high-dose protocol increases the penetration of Lipodox into the brain tissue, likely leading to the enhanced therapeutic efficacy observed in the HD-HFIRE+DOX group. However, as Lipodox concentration following low-dose H-FIRE was not investigated, we may hypothesize that sufficient Lipodox did not enter the brain with this protocol. It is crucial to ensure that the lower-dose protocol is also effective, as it may offer several advantages, including reduced toxicity and improved patient tolerance. Therefore, conducting both high-dose and low-dose drug concentration investigations prior to protocol selection will provide crucial insights into the optimal dosing strategy for Lipodox in the treatment of brain tumors.

Furthermore, our studies demonstrate that H-FIRE can stimulate immune cell infiltration within the tumor microenvironment. H-FIRE-mediated immune responses were assessed *via* IHC performed on treated tissue sections, serum cytokine analysis, and gene expression and pathway analysis. Our results suggest that H-FIRE may be capable of counteracting the immunosuppressive effects of the tumor microenvironment by stimulating pro-inflammatory local and systemic immune responses *via* P/DAMP signaling. In addition, H-FIRE-induced BBB disruption may further facilitate the entry of immune cells into the tumor and activation of the adaptive immune system, which has the potential to induce an anti-tumor response and enhance local tumor control. Additionally, these findings suggest that H-FIRE ablation induces a pro-inflammatory response characterized by the release of various cytokines, which may contribute to tissue damage and regeneration.

The applications of electroporation for BBB disruption are expanding and have demonstrated the potential to further enhance our studies. It is important to note that the mechanistic phenomena of electroporation induced BBB disruption is predominantly due to a loss of tight junction protein integrity caused by endothelial cell cytoskeletal remodeling rather than pore formation in the cellular membrane (37). Studies have suggested that fine tuning electrode configurations as well as the waveform to take on short pulse widths and larger delays may shift toward maximizing BBB disruption field distributions (36). Notably, doxorubicin alone did not improve rodent survival; however, when combined with H-FIRE therapies, improved outcomes were observed, implying that temporary BBB disruption allows for more targeted drug diffusion, potentially reducing the extent of systemic toxicity. Thus, protocols striving for larger BBB disruption may promote improved efficacy of adjuvant liposomal doxorubicin diffusion, allowing for effective targeting of infiltrative cells that have migrated beyond the primary bulk tumor.

Other results have demonstrated the feasibility of applying non-invasive plate electrodes to induce BBB disruption and should be evaluated for combinatorial efficacy (38). These techniques for inducing BBB disruption present the potential for treating not just tumors but also leveraging the power of selectively disrupting the BBB without ablation while still effectively delivering drugs and improving the immune response following the H-FIRE treatments. Leveraging such advantages is not limited to treating gliomas and can be utilized to treat other neurological diseases as well.

Other non-invasive approaches for disrupting the BBB include utilizing focused ultrasound (FUS) in conjunction with microbubbles, however, the skull presents a major challenge for integration of FUS procedures in the brain, as it results in rapid attenuation of sound waves which increases the temperature of the brain limiting the range of safe energy exposures that can be achieved. Additionally, BBB recovery following FUS typically occurs within a few hours, potentially limiting practical treatment windows. Alternatively, convection-enhanced delivery (CED) offers another potential solution for intracranial drug delivery by bypassing the BBB altogether using a hollow catheter placement directly into the target tissue, and using pressure driven flow to deliver a variety of therapeutic agents. Though effective, CED is limited by the occurrence of perfusate reflux and by the requirement for lengthy treatment sessions due to relatively slow infusion rates. The combination of H-FIRE in conjunction with CED may offer the ability to maximize the success of total therapeutic delivered to the target tissue, as H-FIRE may increase permeability of the targeted tissue allowing for the tissue to absorb therapeutics that would have otherwise been lost to the reflux.

We have demonstrated that H-FIRE monotherapy improves the infiltration of immune cells in the tumor microenvironment. H-FIRE treated tumors have shown higher population of T cells, B cells, and microglia. Out of different T cell subtypes, the cytotoxic T cells have shown an improved infiltration in the analyzed sections. The presence of the immune cells was also analyzed with proximity to the tumor mass. Interestingly, the B cells were found to be the most abundant in the tumor mass. The majority of the other immune cells were found in the transition zone. It is noteworthy that the differences observed here are only analyzed at the endpoint. Since the immune response is a dynamic phenomenon, immune cell abundance may have a different profile at other time points.

For the numerical modeling portion of this study, the induced tumor was assumed to take on the shape of a sphere, where treatment plans were designed to encapsulate the estimated 12mm^3^ sphere which was approximated from the tumor growth study. However, it is seen from the histology images (**Figure 6B**) that the tumors grow in an elliptical fashion rather than spherical. We expect that even if the tumor were to deviate from a perfectly spherical shape, the majority of the tumor would still have been encapsulated within the treatment fields due to the establishment of electrode insertion tracks prior to tumor implantation. The tumor was implanted bisecting the electrode insertion track and placed at the midline of the electrode insertion depth. To this fact, the timepoint of 7 days following tumor implant was suitable for investigation of H-FIRE was chosen because further timepoints would necessitate insertion of ≥ 3 needle electrodes.

From the rodent survival study, we were able to observe that the inclusion of H-FIRE as either a monotherapy or a combinatorial therapy does improve the median survival and overall survival of tumor bearing rodents. While significantly increased median survival (p=0.0312) was only seen comparing all rodents receiving H-FIRE therapy (high-dose H-FIRE, low-dose H-FIRE, high-dose H-FIRE+DOX, low-dose H-FIRE+DOX) against the sham group, we note that the highest median survivals were seen in the low-dose H-FIRE, combinatorial high-dose H-FIRE+DOX, and high-dose H-FIRE cohorts, which did not demonstrate statistical significance against the sham group. Therefore, we may infer that low enrollment numbers in each individual cohort contributed to a lack of statistical significance between other groups.

## 5 Conclusion

Expanding on first-generation IRE therapy brain studies, this study has demonstrated the efficacy of applying H-FIRE as both a monotherapy and as a combinatorial (liposomal doxorubicin) therapy for the treatment of brain tumors. Our results show that H-FIRE mediation in the treatment of rat gliomas significantly improves the median survival while demonstrating a potential for greater overall survival when leveraging it as a combinatorial therapy. While H-FIRE can be effectively used as a monotherapy to target the tumor on its own, the surrounding volumes of BBB disruption that facilitate drug delivery into the brain and the reversible electroporation regions that enhance drug uptake into the cells may further enhance the therapeutic effects. The next step toward the treatment of human patients should involve research into single-needle insertion devices capable of producing large transient BBB disruption.

## Data availability statement

The raw data supporting the conclusions of this article will be made available by the authors, without undue reservation.

## Ethics statement

The animal study was reviewed and approved by Institutional Animal Care and Use Committee (#19-217).

## Author contributions

Conceptualization: SC, ML, BP, NA, YK, JH, SV, RD and JR; Data curation: SC, ML, BP, NA, YK, JG, SS, ST, JH, SV, RD and JR; Formal analysis: SC, ML, BP, NA, RD and JR; Funding acquisition: SV, RD and JR; Investigation: SC, ML, BP, NA, YK, JG, SS, ST, JH, SV, RD and JR; Methodology: SC, ML, BP, YK, ST, JH, SV, RD and JR; Project administration: SC, ML, BP, NA, YK, JH, SV, RD and JR; Resources: SC, ML, BP, NA, YK, JH, SV, RD and JR; Software: SC and ML; Supervision: SC, ML, BP, RD and JR; Validation: SC, ML, BP, NA, RD and JR; Visualization: SC, ML, BP, NA, RD and JR; Writing – original draft: SC and ML; Writing - review and editing: SC, ML, BP, NA, YK, JG, SS, ST, JH, SV, RD and JR; All authors contributed to the article and approved the submitted version.
