# Supplementary material for: High-frequency irreversible electroporation improves survival and immune cell infiltration in rodents with malignant gliomas
Source: Front Oncol. 2023 May 5;13:1171278. doi: 10.3389/fonc.2023.1171278 (PMC10196182; doi:10.3389/fonc.2023.1171278)

Rodent Neurologic Severity Score (RNSS)

Assessments		Score			
**Category**	**Test Description**	**Normal** ⟶			**Severe disability**
		**0**	**1**	**2**	**3**
**Spontaneous activity and involuntary movements**	**Cage Observation (1 minute)**	Moves around enclosure and explores walls	Myoclonus or other involuntary movements	Seizures	Unable to move; Recumbent
	**Climbing (3 attempts)**	Readily reaches top of enclosure on first attempt	Trunk twisting/tilting or asymmetric grip requires multiple attempts to reach top	Tends to circle instead of climb	Makes no attempt to reach top of enclosure
**Limb movement and symmetry**	**Tail Suspension Test**	Hindlimbs splayed (extended) laterally	One hindlimb partially extended/clasped	Both hindlimbs partially extended/clasped	Both hindlimbs paralyzed (no joint movement or clasped under midline)
	**Wheelbarrow**	Walks symmetrically on both forelimbs	One limb extends less than the other or deviates to one side (specify limb)	One forelimb paralyzed (specify limb)	Both forelimbs paralyzed (no joint movement)
	**Open Field Gait Analysis**	Walks normally	Unable to walk in straight line	Circling or falling to one side (specify side of deficit)	Unable to advance limbs during gait (specify affected limbs)
**Sensory evaluation**	**Vibrissae touch**	Symmetric response (turns head towards stimulus or is startled)	Slow response on one or both sides (specify side of deficit)	No response on one side (specify side of deficit)	No response on both sides

A total RNSS is assigned after test battery completion, ranging from 0 to 18.

Total RNSS	Disease Severity
**0**	Normal; subclinical disease
**1-6**	Mild disease
**6-12**	Moderate disease
**12-18**	Severe disease (humane endpoint; consult protocol)

Tail Suspension Test

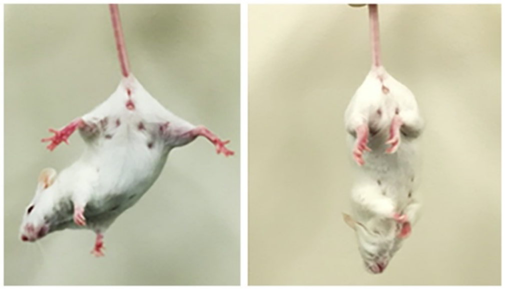

Normal = Score 0 **Severe = Score 3**

Wheelbarrow

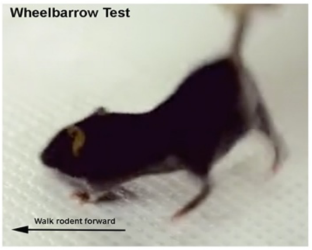

Supplement: Supplementary file 1 [file DataSheet_1.pdf]
