# Supplementary material for: High-frequency irreversible electroporation improves survival and immune cell infiltration in rodents with malignant gliomas
Source: Front Oncol. 2023 May 5;13:1171278. doi: 10.3389/fonc.2023.1171278 (PMC10196182; doi:10.3389/fonc.2023.1171278)
Supplement: Supplementary file 2 [file DataSheet_2.pdf]

Treatment Protocols

Ablation Protocol: → + Lipodox

- 5-5-5 μs, 200 bursts, 100 μs on-time
- Voltage/distance: 1,750 V/cm (525 V)

1 hr BBB disruption coverage: 100%

H-FIRE ablation coverage: 98.7%

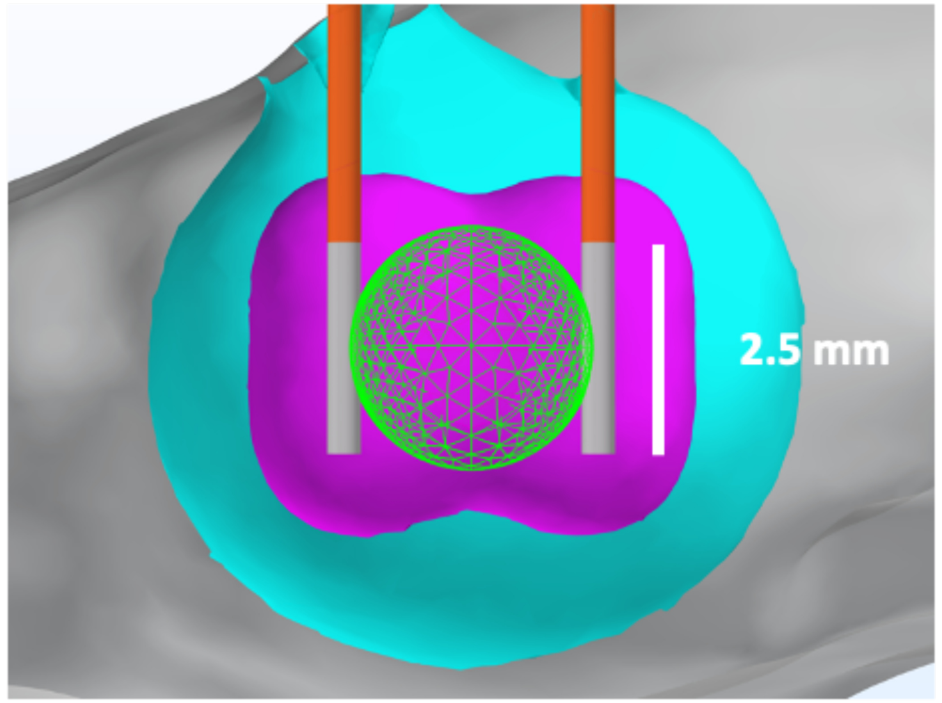

Front view

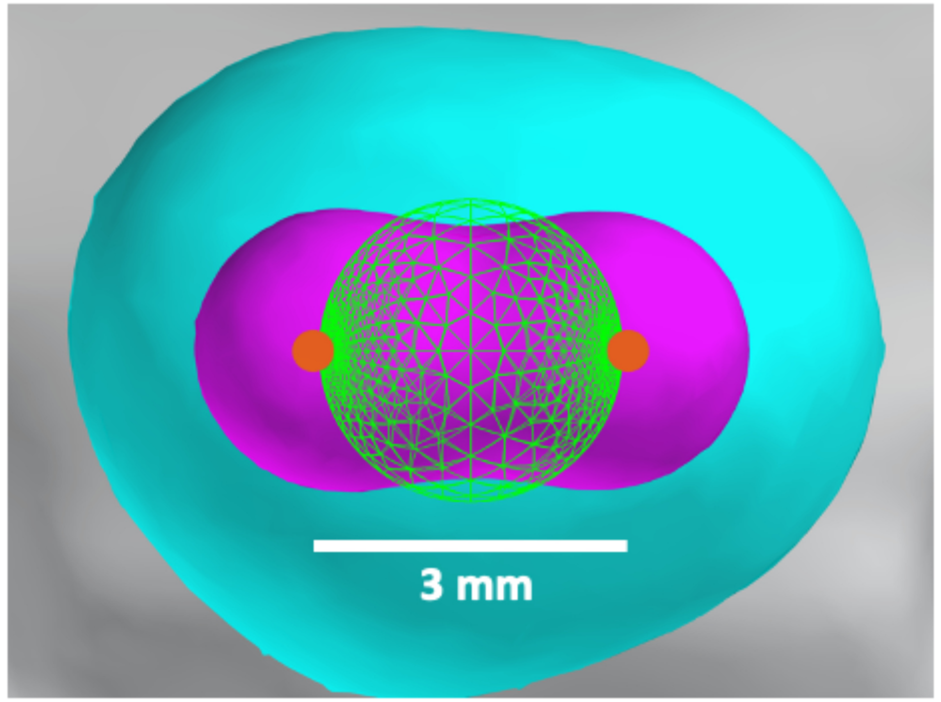

Top view

BBBD Only Protocol: → + Lipodox

- 5-5-5 μs, 200 bursts, 100 μs on-time
- Voltage/distance: 600 V/cm (180 V)

1 hr BBB disruption coverage: 100%

24 hr BBB disruption coverage: 100%

H-FIRE ablation coverage: 6.7%

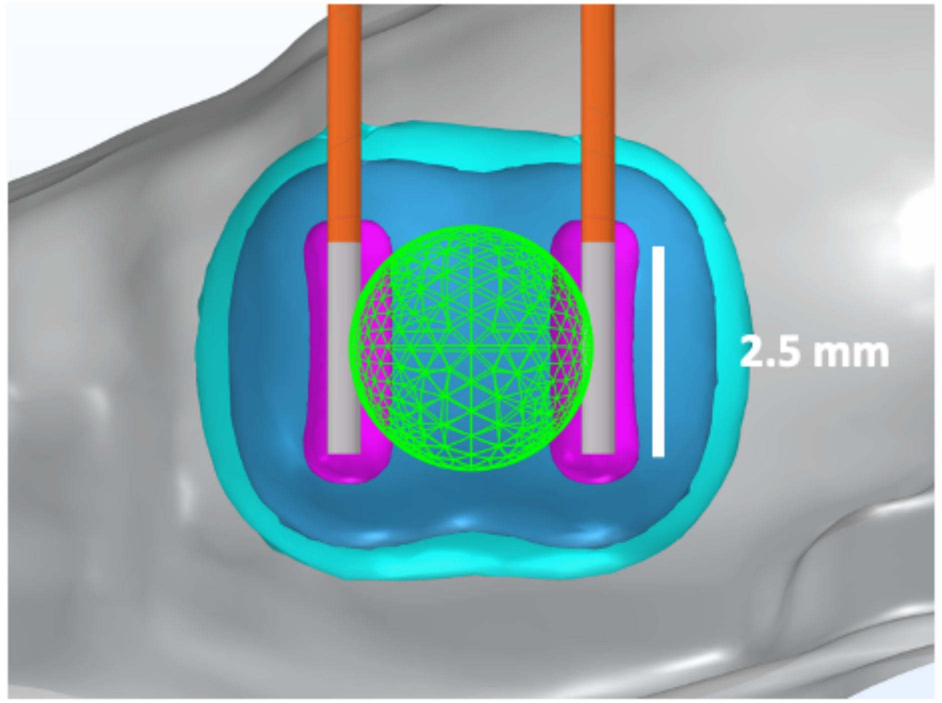

Front view

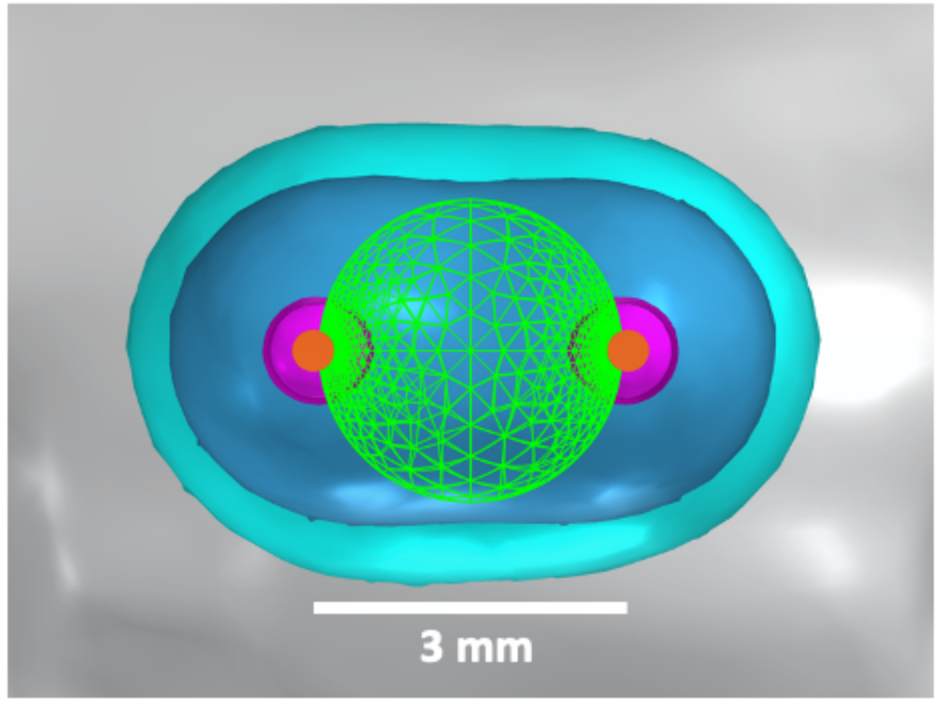

Top view
